# Optimal design of proportional–integral controllers for stand-alone solid oxide fuel cell power plant using differential evolution algorithm

**DOI:** 10.1186/s40064-016-2025-8

**Published:** 2016-03-31

**Authors:** Ashik Ahmed, Md. Shahid Ullah

**Affiliations:** EEE Department, Islamic University of Technology, Boardbazar, Gazipur Bangladesh

**Keywords:** Solid oxide fuel cell, Differential evolution algorithm, Small signal model, Eigen-value based objective function, Particle swarm optimization algorithm and invasive weed optimization algorithm

## Abstract

This paper proposes the application of differential evolution (DE) algorithm for the optimal tuning of proportional–integral (PI) controller designed to improve the small signal dynamic response of a stand-alone solid oxide fuel cell (SOFC) system. The small signal model of the study system is derived and considered for the controller design as the target here is to track small variations in SOFC load current. Two PI controllers are incorporated in the feedback loops of hydrogen and oxygen partial pressures with an aim to improve the small signal dynamic responses. The controller design problem is formulated as the minimization of an eigenvalue based objective function where the target is to find out the optimal gains of the PI controllers in such a way that the discrepancy of the obtained and desired eigenvalues are minimized. Eigenvalue and time domain simulations are presented for both open-loop and closed loop systems. To test the efficacy of DE over other optimization tools, the results obtained with DE are compared with those obtained by particle swarm optimization (PSO) algorithm and invasive weed optimization (IWO) algorithm. Three different types of load disturbances are considered for the time domain based results to investigate the performances of different optimizers under different sorts of load variations. Moreover, non-parametric statistical analyses, namely, one sample Kolmogorov–Smirnov (KS) test and paired sample *t* test are used to identify the statistical advantage of one optimizer over the other for the problem under study. The presented results suggest the supremacy of DE over PSO and IWO in finding the optimal solution.

## Background

Advancements in science and technology pave the way of application of modern sophisticated devices which require more electricity. The fossil fuel based energy resources are diminishing at a rapid rate which makes it incumbent to search for sustainable energy sources. Two of the mostly used renewable energy sources namely, wind and solar have their major drawbacks in terms of constancy in supply throughout any specific period of study. At the same time, installation of newer transmission lines to increase transfer of power to remote places is not encouraged due to lack of land access. With the introduction of the concept of distributed generation (DG), a new paradigm has been opened in the field of electric power system research. The DGs possess exciting features like: reduced transmission loss, less dependence on fossil fuel, steady in supply and clean in nature (Pepermans et al. 2005). Among the available DG sources fuel cells (FCs) have observed one the highest level of application in electricity generation. Solid oxide fuel cell (SOFC), one member of the FC family, has attracted much attention recently as one of the most efficient electricity producing device (Choudhury et al. 2013). Application of SOFC in both stand alone and grid connected modes are reported in the literature. The stand-alone SOFCs are finding their applications in powering cars, small homes and isolated or distant areas.

Different dimensional modeling of SOFC has been reported in Nehter (2006), Recknagle et al. (2003) and Bove et al. (2005). These models are mostly based on the internal mechanism of the SOFC which has little to do with the control design of external parameters. Artificial neural network (ANN) is used in Arriagada (2002) to predict the performance of SOFC stack. An adaptive network based fuzzy-inference system (ANFIS) is proposed in Wu et al. (2008) to replicate the SOFC performance. The T–S fuzzy model based SOFC system identification is presented in Wu et al. (2008). In all these data driven based approaches independent control of SOFC variables are considered. To overcome this shortcoming a radial basis function (RBF) based hybrid model of SOFC is proposed in Wu et al. (2009) where a performance index is defined to evaluate the modeling accuracy.

Once a suitable dynamic model has been established, it becomes customary to conduct the control relevant studies of a physical system. A model predictive controller for a rigorous SOFC dynamic model has been proposed in Jacobsen et al. (2013). Although the proposed controller shows good control effort, the understanding is difficult to grasp and implementation in practice is complicated. A constant fuel utilization control is presented in Li et al. (2005) where a feasible operating area (FOA) of SOFC operation is identified and a small signal model based controller is designed to keep the operation within FOA under different sorts of load disturbances. The dynamic model of SOFC considered does not include the temperature dynamics and thus may not give the most accurate transient behavior. An observer based transient control of SOFC fuel utilization is depicted in Das and Slippey (2010). The efficacy of this approach is mostly based on the accuracy of the estimated data and a small error in measurement might yield unsatisfactory behavior of the control effort. Data driven nonlinear controller for SOFC is proposed in Li et al. (2012) where combination of support vector machine (SVM) and virtual reference feedback tuning (VRFT) method is utilized to solve the SOFC control problem. However, as this combination failed to ensure safe operation of SOFC, a feed-forward loop had been incorporated. An FPGA based dc–dc converter controller is presented in Bhuyan et al. (2013) which control the output voltage of SOFC by injecting proper PWM signals. Simple PI regulator is used to track the error between the reference and actual values of SOFC voltage output. In this case the control is provided through the power electronic interface instead of the fuel flow control and as a result the fuel utilization may go beyond the limit. The Matlab/Simulink based dynamic model is used to simulate and study the dynamic behavior of SOFC in Kamel et al. (2013). It is reported that the stand-alone SOFC is suitable to be used in a grid system with DGs but shows relatively sluggish response to external disturbances. The focus of the work was not to design efficient controller but to present comparative study among different types of DGs.

From the previous works presented above it may be summarized that (1) either the SOFC dynamic model used are too complex and includes such detail which might seem too involved for the users or the models are built ignoring important dynamics (i.e. temperature dynamics) which forms one of the integral part of the SOFC physical system, (2) control design is either too complicated which makes it difficult to be implemented in practice or done at the power electronics interface without keeping the limit of fuel utilization in mind which might result reduced lifetime of the cell. Keeping these observations in mind this paper adopts such a dynamic model of the SOFC which is not too complex and at the same time incorporates all the basic dynamic such as, partial pressure dynamics of participating gases (Padullés et al. 2000), temperature dynamics and fuel cell electrical dynamics (Du et al. 2012). The inclusion of the temperature dynamics modifies the species dynamics and the SOFC output voltage expressions a little. As the target in this work is to design controllers which should work well under small disturbance, the overall system model is linearized using Taylor series expansion technique (Ghilani 2010) and the disturbance is simulated as small variations in the load. The controllable parameters of the stand-alone SOFC under consideration are the partial pressure of the hydrogen and the oxygen gases. These two are controlled using their respective inlet molar flow rates at the anode and cathode chambers of the SOFC. The linearized closed loop system matrix is formed whose eigenvalues are dependent on the PI controller parameters. An eigenvalue based objective function is considered and the controller gains are optimized with the help of differential evolution (DE) algorithm which has already been applied successfully in solving different engineering optimization problem (Wang et al. 2009; Abou El Ela et al. 2009; Qin et al. 2009; Vesterstrom and Thomsen 2004). Performance of DE is then compared to those of PSO and IWO to show the efficacy of DE in solving the problem under consideration. Eigen-value based study, time domain based simulation and non-parametric statistical analyses are utilized to compare the performances of the optimizers.

The mathematical model of the stand-alone SOFC is presented in second section. Third section discusses the linearization of the model based on the Taylor series expansion and includes the open loop responses. A detail description of the DE algorithm, controller configuration, the closed loop system formation and formulation of the optimization problem is discussed in fourth section. The eigenvalue based study, time domain based simulation results and statistical analyses outcome showing the effectiveness of the DE over PSO and IWO are presented in fifth section and lastly the paper is concluded in sixth section.

## Mathematical model of stand-alone SOFC

**Fig. 1:**
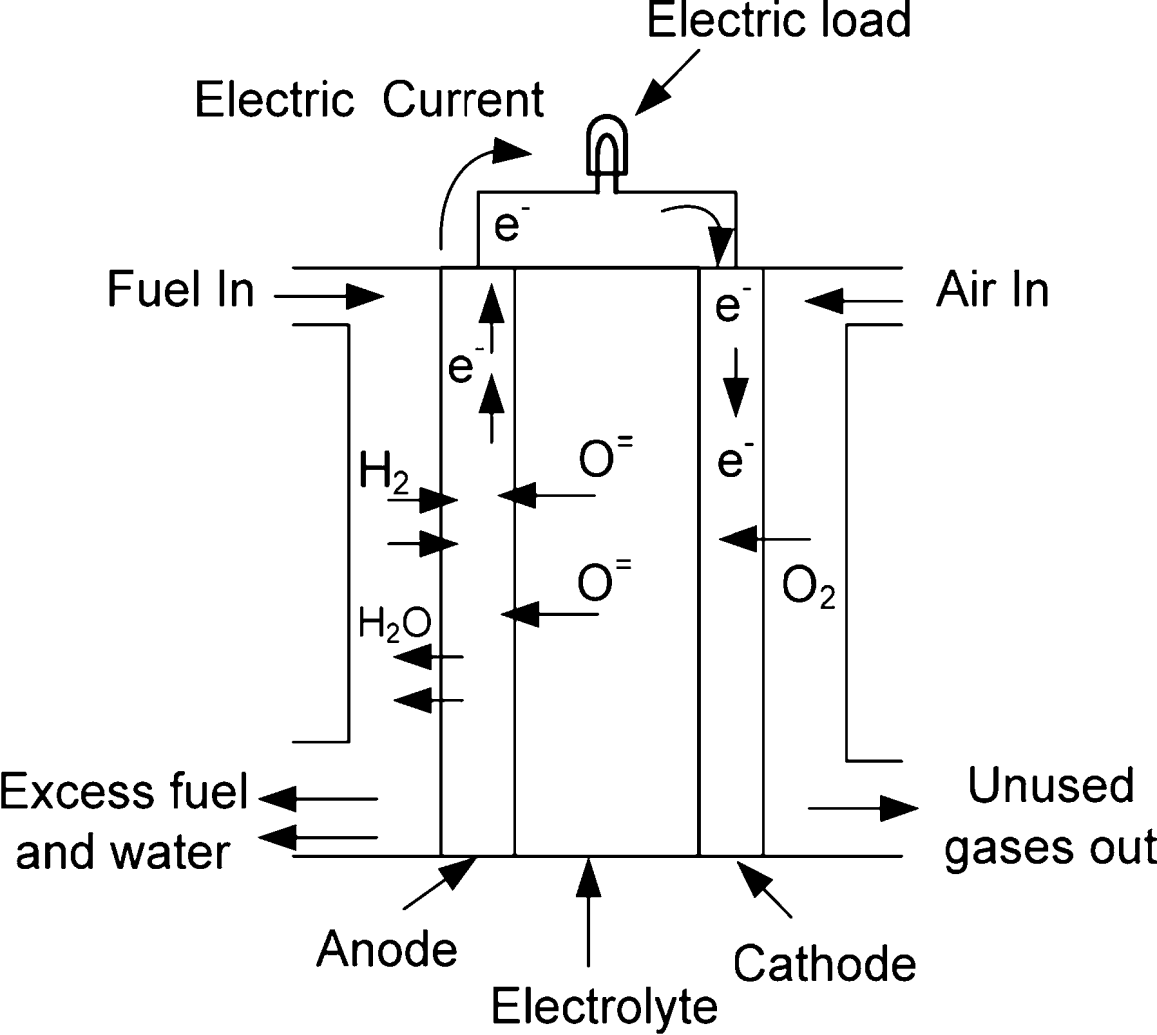
Schematic of solid oxide fuel cell

The basic operation of a single SOFC is demonstrated in Fig. 1 which follows the reduction–oxidation (Red–Ox) reaction of Eqs. (1–3).

1 $${\text{Cathode}}\;{\text{reaction}}\;\left( {{\text{reduction}}\;{\text{of}}\;{\text{oxygen}}} \right){:}\;\frac{1}{2}O_{2} + 2e^{ - } \to O^{2 -}$$

2 $${\text{Anode}}\;{\text{reaction}}\; ( {\text{oxidation}}\;{\text{of}}\;{\text{hydrogen)}}{:}\;H_{2} + O^{2 - } \to H_{2} O + 2e^{ - }$$

3 $${\text{Overall}}\;{\text{reaction}}{:}\;H_{2} + \frac{1}{2}O_{2} \to H_{2} O$$

The cell output voltage of a SOFC stack with *N*_0_ cells in series can be expressed by Nernst equation:

4 $$V = N_{0} E_{0} + \frac{{N_{0} RT}}{2F}\ln \left( {\frac{{P_{{H_{2} }} P_{{O_{2} }}^{0.5} }}{{P_{{H_{2} O}} }}} \right)$$

Here, $P_{{H_{2} }}$, $P_{{O_{2} }}$ and $P_{{H_{2} O}}$ are the partial pressures of the hydrogen, oxygen and water vapor respectively. *R* and *T* are the universal gas constant and electrode temperature expressed in J/(mol-°C) and kelvin, respectively. The temperature dependence of *E*_0_ can be expressed as (Campanari and Iora 2004): *E*_0_ = *E*_*noloss*_ − 0.000252 * *T*, where *E*_*noloss*_ is the theoretical maximum voltage of the cell. The ohmic drop *E*_*ohmic*_, under loaded condition can be represented as a function of cell operating temperature as (Sedghisigarchi and Feliachi 2004):

5 $$E_{ohmic} = r_{0} \exp \left[ {\alpha \left( {\frac{1}{T} - \frac{1}{{T_{0} }}} \right)} \right]I_{fc}$$

Here, *r*_0_: resistance at standard temperature *T*_0_, *α*: constant coefficient, *I*_*fc*_: cell current at the operating temperature *T*. Inclusion of this loss modifies the expression of cell voltage as:

6 $$V_{s} = N_{0} E_{0} + \frac{{N_{0} RT}}{2F}\ln \left( {\frac{{P_{{H_{2} }} P_{{O_{2} }}^{0.5} }}{{P_{{H_{2} O}} }}} \right) - r_{0} \exp \left[ {\alpha \left( {\frac{1}{T} - \frac{1}{{T_{0} }}} \right)} \right]I_{fc}$$

The SOFC power can simply be expressed as:

7 $$P_{sofc} = V_{s} I_{fc}$$

Equations (6–7) governs the steady state characteristics of a SOFC.

### Dynamic modeling of stand-alone SOFC

Two major balances are to be considered to represent the overall dynamics of an SOFC. These are—component material balance and energy balance. The component material balance represents the change in balance which occurs in different species during the chemical reaction in an SOFC whereas the energy balance ensures the equilibrium between associated input and output energy levels.

### Component material balance

The partial pressure of the *i*-th species of a SOFC can be written as:

8 $$\dot{P}_{i} = \frac{T}{{\tau_{i}^{0} T^{0} K_{i} }}\left( {n_{i}^{in} - n_{m} K_{r} I_{fc} - K_{i} P_{i} } \right)$$

Here, *τ*^0^: time constant at temperature *T*^0^, ${n}_{i}^{in}$: inlet molar flow rates for *i*-th species, *n*_*m*_: number of moles present in the reaction, *K*_*r*_ = *N*_0_/(*4F*), *K*_*i*_, *P*_*i*_: valve molar constant and partial pressure of *i*-th specie. Defining $\tau_{i} = \frac{{\tau_{i}^{0} T^{0} }}{T}$, from the generalized relation of Eq. (8) the partial pressure dynamics for different species of SOFC can be expressed as:

9 $$\dot{P}_{{H_{2} }} = \frac{T}{{\tau_{{H_{2} }}^{0} T^{0} K_{{H_{2} }} }}\left( {n_{{H_{2} }}^{in} - 2K_{r} I_{fc} - K_{{H_{2} }} P_{{H_{2} }} } \right)$$

10 $$\dot{P}_{{O_{2} }} = \frac{T}{{\tau_{{O_{2} }}^{0} T^{0} K_{{O_{2} }} }}\left( {n_{{O_{2} }}^{in} - K_{r} I_{fc} - K_{{O_{2} }} P_{{O_{2} }} } \right)$$

11 $$\dot{P}_{{H_{2} O}} = \frac{T}{{\tau_{{H_{2} O}}^{0} T^{0} K_{{H_{2} O}} }}\left( {n_{{H_{2} O}}^{in} + 2K_{r} I_{fc} - K_{{H_{2} O}} P_{{H_{2} O}} } \right)$$

The *n*_*m*_ values for different species are obtained from the basic SOFC red–ox Eqs. (1–3).

### Energy balance

The energy balance deals with various heat transfers occurring at different layers of an SOFC. If lumped model of SOFC is considered, application of first law of thermodynamics around the entire SOFC yields the following dynamics of electrode temperature, T

12 $$m_{e} \bar{C}_{p} \frac{dT}{dt} = \sum {{n}_{i}^{in} \int\limits_{{T_{ref} }}^{{T_{in} }} {C_{p,i} } } \left( T \right)dT - \sum {{n}_{i}^{out} \int\limits_{{T_{ref} }}^{T} {C_{p,i} } } \left( T \right)dT - {n}_{{H_{2} }}^{r} \Delta \hat{H}_{r}^{o} - V_{s} I$$

where *m*_*e*_ and $\bar{C}_{p}$ are the mass and average specific heat of the cell excluding gases; *C*_*p*,*i*_ is the specific heat of the *i*-th fuel or gas entering or leaving the cell; $\Delta \hat{H}_{r}^{o}$ is the specific heat of reaction and *V*_*s*_ is the cell stack voltage. The expressions of specific heats are usually adopted from standard thermodynamics table (Coker 1995) and in general expressed as, $C_{p,i} (T) = a_{i} + b_{i} T + c_{i} T^{2} + d_{i} T^{3}$.

### SOFC electrical dynamics

The electrical dynamics represent the chemical reaction inside the SOFC to restore the charge which has been used up by the load. A first-order transfer function is used to model the dynamic with the time constant *T*_*el*_. The differential form of this dynamics can be expressed as:

13 $$\dot{I}_{fc} = \frac{1}{{T_{el} }}\left( {I_{fcref} - I_{fc} } \right)$$

Here, *I*_*fcref*_ is the reference current of the SOFC. The ODEs of Eqs. (9–13) and the algebraic Eqs. (6–7) form the complete differential–algebraic model of the stand-alone SOFC.

### System description

**Fig. 2:**
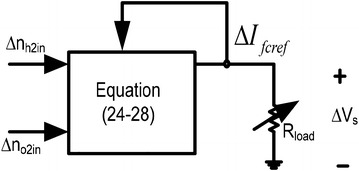
System configurations in open loop

The system under consideration is presented in Fig. 2. *R*_*load*_ is a variable resistive load connected at the terminals of SOFC which dictates the changes in SOFC reference current. Any change in the reference current is followed by variations in the actual cell current. Variations in the actual cell current affect the reaction rates of the SOFC which in turn changes the partial pressures of the cell species. The cell performance will deteriorate if proper control measures are not taken for partial pressures. The partial pressures can be controlled by manipulating the input flow rates as seen from Eqs. (9–11). In practice, only the hydrogen and oxygen flow rates can be utilized for regulating the respective partial pressures. So, the SOFC plant can be considered as a two-input, two-output system with partial pressures of hydrogen and oxygen are the outputs to be controlled and the respective flow rates are the manipulated inputs. If only small variations in the load are considered, linearized model of the stand-alone SOFC system can be derived and employed for the control design purpose. As study of eigenvalues of a linearized system gives an indication of the amount of control achieved, the controller design problem is formulated as optimization of an eigenvalue based objective function which depends on the numerical values of the controller gains. PI controllers are used in this work because of the versatility and easiness in real life implementation and the gains of the controllers are optimized with the help of DE algorithm.

## Linearization of SOFC dynamic model and open loop response

The SOFC dynamic model developed in section “Mathematical model of stand-alone SOFC” can be represented in state space form as $\varvec{\dot{\bar{x}}} = \varvec{f}(\bar{\varvec{x}}) + \varvec{g}(\bar{\varvec{x}}) \cdot \varvec{u}$, where $\varvec{f}(\bar{\varvec{x}})$ and $\varvec{g}(\bar{\varvec{x}})$ are nonlinear functions of the state vector, $\bar{\varvec{x}} = [P_{{h_{2} }} \;P_{{o_{2} }} \;P_{{h_{2} o}} \;T\;I_{fc} ]$ and *u* is the control vector, $\varvec{u} = \left[ {n_{{h_{2}}}^{in} \,n_{{o_{2}}}^{in} } \right]$. Using Taylor series expansion and truncating the terms with order 2 and above, the linearized state equations can be obtained as:

14 $$\begin{aligned} \Delta \dot{P}_{{h_{2} }} &= \frac{{ - T^{0} }}{{T_{0} \tau_{{H_{2} }}^{0} }}\Delta P_{{h_{2} }} - \frac{{\left( {K_{{h_{2} }} P_{{h_{2} }}^{0} - n_{{h_{2} }}^{in0} + 2I_{fc}^{0} K_{r} } \right)}}{{K_{{h_{2} }} T_{0} \tau_{{H_{2} }}^{0} }}\Delta T \\&\quad- \frac{{2K_{r} T^{0} }}{{K_{{h_{2} }} T_{0} \tau_{{H_{2} }}^{0} }}\Delta I_{fc} + \frac{{T^{0} }}{{T_{0} K_{{h_{2} }} \tau_{{H_{2} }}^{0} }}\Delta n_{{h_{2} }}^{in} \end{aligned}$$

15 $$\begin{aligned} \Delta \dot{P}_{{O_{2} }}& = \frac{{ - T^{0} }}{{T_{0} \tau_{{O_{2} }}^{0} }}\Delta P_{{O_{2} }} - \frac{{\left( {K_{{O_{2} }} P_{{O_{2} }}^{0} - n_{{O_{2} }}^{in0} + I_{fc}^{0} K_{r} } \right)}}{{K_{{O_{2} }} T_{0} \tau_{{O_{2} }}^{0} }}\Delta T \\&\quad - \frac{{K_{r} T^{0} }}{{K_{{O_{2} }} T_{0} \tau_{{O_{2} }}^{0} }}\Delta I_{fc} + \frac{{T^{0} }}{{T_{0} K_{{O_{2} }} \tau_{{O_{2} }}^{0} }}\Delta n_{{O_{2} }}^{in} \end{aligned}$$

16 $$\Delta \dot{P}_{{h_{2} O}} = \frac{{ - T^{0} }}{{T_{0} \tau_{{h_{2} O}}^{0} }}\Delta P_{{h_{2} O}} - \frac{{\left( {K_{{h_{2} O}} P_{{h_{2} O}}^{0} - 2I_{fc}^{0} K_{r} } \right)}}{{K_{{h_{2} O}} T_{0} \tau_{{h_{2} O}}^{0} }}\Delta T + \frac{{2K_{r} T^{0} }}{{K_{{h_{2} O}} T_{0} \tau_{{h_{2} O}}^{0} }}\Delta I_{fc}$$

17 $$\begin{aligned} \Delta \dot{T} &= T_{ph} \Delta P_{{h_{2} }} + T_{po} \Delta P_{{O_{2} }} + T_{pw} \Delta P_{{h_{2} O}} + T_{pt} \Delta T \\&\quad + T_{pi} \Delta I_{fc} + T_{pnh} \Delta n_{{h_{2} }}^{in} + T_{pno} \Delta n_{{O_{2} }}^{in} \end{aligned}$$

18 $$\Delta \dot{I}_{fc} = \frac{1}{{T_{el} }}\left( {\Delta I_{fcref} - \Delta I_{fc} } \right)$$

Detail expressions of *T*_*ph*_, *T*_*po*_, *T*_*pw*_, *T*_*pt*_, *T*_*pi*_, *T*_*pnh*_ and *T*_*pno*_ are given in the “Appendix”. If it is desired to represent the Eqs. (14–18) in the form $\Delta \varvec{\dot{\bar{x}}} = A\Delta \bar{\varvec{x}} + B\Delta \varvec{u}$, the matrices *A* and *B* are found as:

$$A = \left[ {\begin{array}{*{20}c} {\frac{{ - T^{0} }}{{T_{0} \tau_{{H_{2} }}^{0} }}} & 0 & 0 & { - \frac{{\left( {K_{{h_{2} }} P_{{h_{2} }}^{0} - n_{{h_{2} }}^{in0} + 2I_{fc}^{0} K_{r} } \right)}}{{K_{{h_{2} }} T_{0} \tau_{{H_{2} }}^{0} }}} & { - \frac{{2K_{r} T^{0} }}{{K_{{h_{2} }} T_{0} \tau_{{H_{2} }}^{0} }}} \\ 0 & {\frac{{ - T^{0} }}{{T_{0} \tau_{{O_{2} }}^{0} }}} & 0 & { - \frac{{\left( {K_{{h_{2} O}} P_{{h_{2} O}}^{0} - 2I_{fc}^{0} K_{r} } \right)}}{{K_{{h_{2} O}} T_{0} \tau_{{h_{2} O}}^{0} }}} & { - \frac{{K_{r} T^{0} }}{{K_{{O_{2} }} T_{0} \tau_{{O_{2} }}^{0} }}} \\ 0 & 0 & {\frac{{ - T^{0} }}{{T_{0} \tau_{{h_{2} O}}^{0} }}} & { - \frac{{\left( {K_{{h_{2} O}} P_{{h_{2} O}}^{0} - 2I_{fc}^{0} K_{r} } \right)}}{{K_{{h_{2} O}} T_{0} \tau_{{h_{2} O}}^{0} }}} & {\frac{{2K_{r} T^{0} }}{{K_{{h_{2} O}} T_{0} \tau_{{h_{2} O}}^{0} }}} \\ {T_{ph} } & {T_{po} } & {T_{pw} } & {T_{pt} } & {T_{pi} } \\ 0 & 0 & 0 & 0 & {\frac{{ - \Delta I_{fc} }}{{T_{el} }}} \\ \end{array} } \right]$$

$$B = \left[ {\begin{array}{*{20}c} {\frac{{T^{0} }}{{T_{0} K_{{h_{2} }} \tau_{{H_{2} }}^{0} }}} & 0 & 0 & {T_{pnh} } & 0 \\ 0 & {\frac{{T^{0} }}{{T_{0} K_{{O_{2} }} \tau_{{O_{2} }}^{0} }}} & 0 & {T_{pno} } & 0 \\ \end{array} } \right]^{\,\,T}$$

### Open loop response

**Fig. 3:**
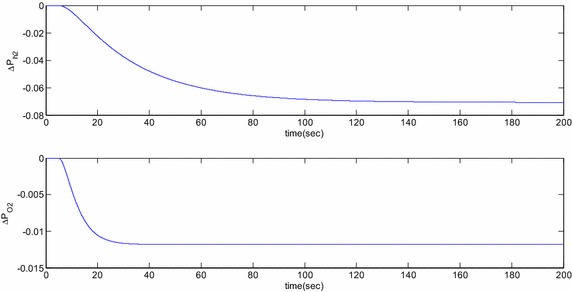
Open loop responses of hydrogen and oxygen partial pressures for a step change in SOFC reference current

**Fig. 4:**
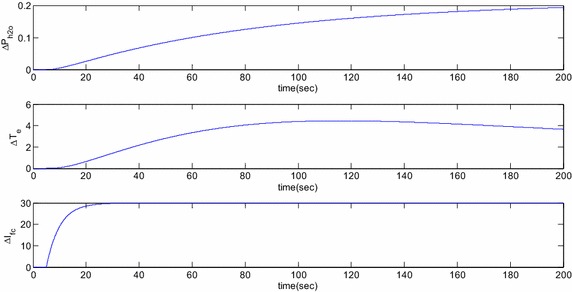
Open loop responses of water vapor partial pressure, temperature and current for a step change in SOFC reference current

Eigenvalue based study and the time domain based simulation results for the open loop response of the SOFC linearized dynamic model is reported here. Numerical data used for these studies are listed in the “Appendix”. Open loop eigenvalues are listed in Table 1 and the corresponding time domain simulations are shown in Figs. 3 and 4 for step change $\Delta I_{fcref}$ = 30 A applied at 5 s. Observation of Table 1 reveals that all eigenvalues of the studied SOFC stand-alone system are real in nature and do not possess any oscillating frequency. Thus, once disturbed, the controllable system states, i.e. the partial pressures of oxygen and hydrogen show sluggish responses. This fact is presented in Fig. 3 where it is found that the system dynamics for these states are stable under this disturbance but the variables settle to new steady state values after long duration owing to large time constants. Specifically, the settling times for the hydrogen and oxygen partial pressures are more than 70 and 20 s, respectively. The participation factor (Sanchez-Gasca et al. 2007) column of Table 1 reveals the fact that all the states are completely decoupled and introduction of control in one of the states will have minimal or no impact on the others. So, if it is desired that the changes in the partial pressures of oxygen and hydrogen are to be tracked by the controllers, status of the other states are going to be mostly unchanged. Figure 4 shows the responses of the remaining three state variables where it is observed that all of them are stable in nature. The water vapor partial pressure and SOFC temperature increases and settles to a new value after a long duration whereas the SOFC current reaches the new equilibrium after 29.76 s. The water vapor partial pressure is measured at the outlet of the SOFC in practice and explicit regulators are not present there. Independent temperature controller is required for proper control of the temperature dynamics but is out of the scope of this work and hence not included.

**Table 1:** Open loop eigenvalues

Eigenvalues	Associated states	Participation factor (%)
−0.0171	∆*T*_*e*_	100
−*0.0383*	$\Delta P_{{h_{2} }}$	100
−*0.3436*	$\Delta P_{{o_{2} }}$	100
−0.0128	$\Delta P_{{h_{2} o}}$	100
−0.2	∆*I*_*fc*_	100

**Fig. 5:**
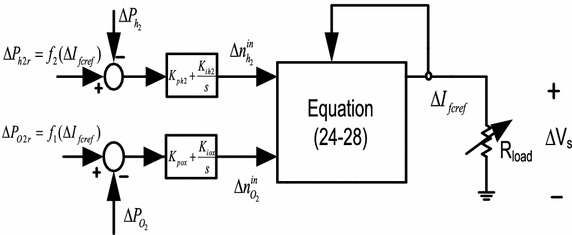
Closed loop stand-alone SOFC system with two PI controllers

To improve the small signal dynamics of hydrogen and oxygen partial pressures, two PI controllers are incorporated in the corresponding feedback loops as depicted in Fig. 5. The inputs to the PI controllers are the error between the actual and reference partial pressures whereas the outputs are the controlled flow rates of hydrogen and oxygen, respectively. The controllers should work in such a way that the system not only remains stable but also shows better transient responses compared to the open loop responses. Gains ${\text{K}}_{{{\text{ph}}_{2} }}$, ${\text{K}}_{{{\text{ih}}_{2} }}$, ${\text{K}}_{{{\text{po}}_{ 2} }}$ and ${\text{K}}_{{{\text{io}}_{2} }}$ are the parameters to be optimized using DE algorithm which is discussed next. For comparison purpose the PSO and IWO are also applied for obtaining the optimal controller gains. Detail discussion on PSO and IWO is not provided here which can be found in Abido (2002) and Chowdhury et al. (2011), respectively.

## Differential evolution and objective function

DE is one of the members of evolutionary algorithm family having attractive feature of solving optimization problem. The main steps in DE algorithm are- initialization of a set of solution, mutation, recombination and selection.

### Initialization

In the initialization phase a random set of probable solution for each parameter is generated within the search space. If an objection function with *D* real parameters is to be optimized for an initial population of size *NP*, the parameters vector takes the form $\varvec{X}_{i,G} = \left[ {\varvec{x}_{1,i,G} ,\varvec{x}_{2,i,G} , \ldots ,\varvec{x}_{D,i,G} } \right]$ with $i=1,2,\ldots,NP$ and *G* is the generation number. With the upper and lower bounds for each parameter defined as $x_{j}^{L} \le x_{j,i,1} \le x_{j}^{U}$, the random parameters in each generation should lie within the interval $\,\left[ {x_{j}^{L} ,x_{j}^{U} } \right]$. As each initialization phase generates different random set of solutions, several runs are to be conducted to obtain the optimal solution for a given problem.

### Mutation

Three target vectors $\varvec{x}_{r1,G} ,\,\,\varvec{x}_{r2,G} ,$ and $\varvec{x}_{r3,G}$ are randomly selected from a given parameter vector $\varvec{X}_{i,G}$ for the mutation phase keeping in mind that the indices *r*1, *r*2, *r*3 and *i* are distinct. These three vectors along with mutation factor *M*_*F*_ are used to generate the donor vector following the strategy *DE*/*rand*/1 as:

19 $$\varvec{v}_{i,G + 1} = \varvec{x}_{r1,G} + M_{F} \left( {\varvec{x}_{r2,G} - \varvec{x}_{r3,G} } \right) .$$

Another way of generating the donor vector $\varvec{v}_{i,G + 1}$ is to follow the *DE*/*Best*/2/*bin* which incorporates four different random vectors plus the best solution of the current generation as:

20 $$\varvec{v}_{i,G + 1} = \varvec{x}_{best,G} + M_{F} \left( {\varvec{x}_{r1,G} + \varvec{x}_{r2,G} - \varvec{x}_{r3,G} - \varvec{x}_{r4,G} } \right)$$

In this work, Eq. (20) is employed for the mutation phase.

### Recombination

In the recombination phase trial vector $u_{j,i,G + 1}$ is generated which gets updated by the donor vector having probability *C*_*R*_.

21 $$u_{j,i,G + 1} = \left\{ {\begin{array}{*{20}l} {v_{j,i,G + 1} } \hfill &\quad {if\;rand_{j,i} \le C_{R} \;or\;j = I_{rand} } \hfill \\ {x_{j,i,G} } \hfill & \quad {if\;rand_{j,i} > C_{R} \;and\;j \ne I_{rand} } \hfill \\ \end{array} } \right.$$

Here, *rand*_*i*,*j*_ is a random number within the range [0, 1] and *I*_*rand*_ is a random integer chosen from $[1,2,\ldots,D]$.

### Selection

In the selection phase a comparison is made between the target vector and trial vector and the ones with the best value is selected and forwarded to the next generation.

22 $$\varvec{x}_{i,G + 1} = \left\{ {\begin{array}{*{20}l} {\varvec{u}_{i,G + 1} } \hfill &\quad {if\;J(\varvec{u}_{i,G + 1} ) \le J(\varvec{x}_{i,G} )} \hfill \\ {\varvec{x}_{i,G} } \hfill &\quad {otherwise} \hfill \\ \end{array} } \right.$$

**Fig. 6:**
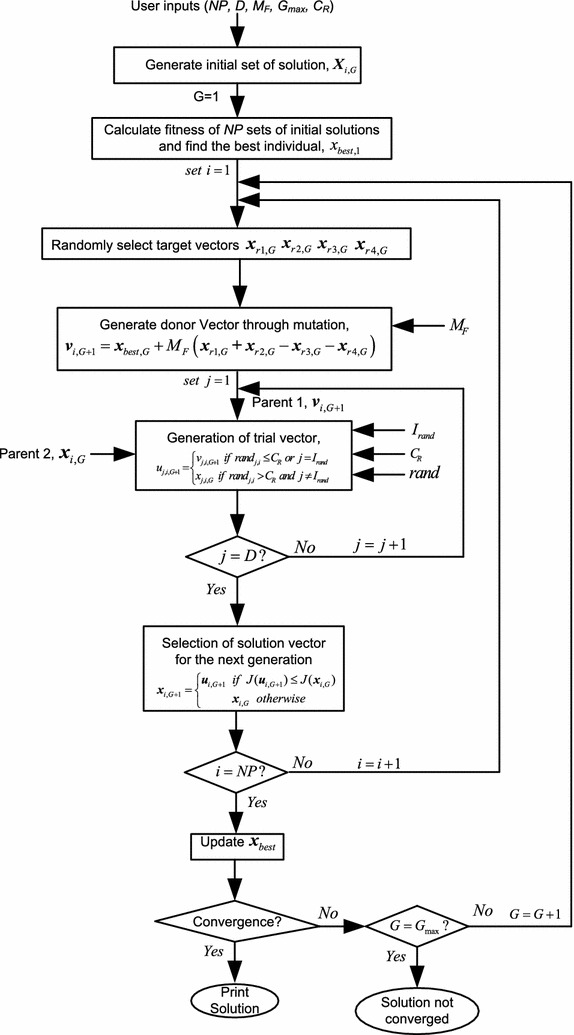
Flowchart of DE algorithm

The mutation, recombination and selection phases continue until a pre-specified stopping criterion is fulfilled. The overall working procedure of the DE algorithm is presented in the flowchart of Fig. 6.

### Objective function

The small signal control of SOFC is initially formulated as a dual-objective optimization problem which is then converted into a single objective function with the help of weighting factor, *w*. DE is employed to minimize the single-objective fitness function which is made up of the eigenvalues of the closed loop system matrix. The closed loop system matrix is again dependent on the PI controller gains as discussed later. The objectives are defined as:

23 $$\begin{aligned} J_{1} & = \sum\limits_{i = 1}^{n} {\left( {\sigma_{i} - \sigma_{0} } \right)^{2} } \\ J_{2} & = \sum\limits_{i = 1}^{n} {\left( {\zeta_{0} - \zeta_{i} } \right)^{2} } \\ J & = (J_{1} + w*J_{2} ) \\ \end{aligned}$$

Here, $\sigma_{i}$ is the real part of the *i*th eigenvalues, $\sigma_{0}$ is the desired value of the real part of the eigenvalues, $\zeta_{i}$ and $\zeta_{0}$ are the actual and desired values of the damping ratios respectively and *w* is the weighting factor which is taken as 0.1 in this work. Optimization of *J*_1_ will ensure that the real parts of the eigenvalues are lying near the desired location and that of *J*_2_ will make sure that sufficient damping has been injected to the system dynamics. So, minimization of *J* will ensure that both *J*_1_ and *J*_2_ objectives are fulfilled simultaneously and an optimized set of controller gains is obtained.

### Reference generation

The change in the reference value of the SOFC current should yield a change in the reference values of the controllable variables, i.e., partial pressures of hydrogen and oxygen. If the left hand sides of Eqs. (9–10) are equated to zero, the resulting steady state relationships in linearized form are obtained as:

24 $$\Delta P_{{h_{2} r}} = - \frac{{ 2K_{r} }}{{K_{{h_{2} }} }}\Delta I_{fcref}$$

25 $$\Delta P_{{O_{2} r}} = - \frac{{ K_{r} }}{{K_{{O_{2} }} }}\Delta I_{fcref}$$

Thus a direct link has been established between the reference values of the cell current and partial pressures.

### Controller configuration and the closed loop system

Once the controllers are incorporated into the system it turns out into a closed loop system which are depicted in Fig. 5. With the introduction of these PI controllers two new state variables are generated as follows:

26 $$\Delta \dot{n}_{{h_{2} }}^{in} = H_{y1} \Delta P_{{h_{2} }} + H_{y2} \Delta T + H_{y3} \Delta I_{fc} + H_{y4} \Delta n_{{h_{2} }}^{in}$$

27 $$\Delta \dot{n}_{{O_{2} }}^{in} = O_{x1} \Delta P_{{O_{2} }} + O_{x2} \Delta T + O_{x3} \Delta I_{fc} + O_{x4} \Delta n_{{O_{2} }}^{in}$$

Detail expressions of the linearizing constants $H_{y1}$, $H_{y2}$, $H_{y3}$, $H_{y4}$, $O_{x1}$, $O_{x2}$, $O_{x3}$ and $O_{x4}$ are listed in the “Appendix” which show that these constants are function of controller gains. The closed loop system in linearized form can be represented as:

28 $$\Delta \varvec{\dot{\bar{x}}} = A_{sys} \Delta \bar{\varvec{x}} + B_{sys} \Delta I_{fcref}$$

The matrices *A*_*sys*_ and *B*_*sys*_ are given as:

$$\begin{aligned} A_{sys} & = \left[ {\begin{array}{*{20}c} {\frac{{ - T^{0} }}{{T_{0} \tau_{{H_{2} }}^{0} }}} & 0 & 0 & { - \frac{{\left( {K_{{h_{2} }} P_{{h_{2} }}^{0} - n_{{h_{2} }}^{in0} + 2I_{fc}^{0} K_{r} } \right)}}{{K_{{h_{2} }} T_{0} \tau_{{H_{2} }}^{0} }}} & { - \frac{{2K_{r} T^{0} }}{{K_{{h_{2} }} T_{0} \tau_{{H_{2} }}^{0} }}} & {\frac{{T^{0} }}{{T_{0} K_{{h_{2} }} \tau_{{H_{2} }}^{0} }}} & 0 \\ 0 & {\frac{{ - T^{0} }}{{T_{0} \tau_{{O_{2} }}^{0} }}} & 0 & { - \frac{{\left( {K_{{O_{2} }} P_{{O_{2} }}^{0} - n_{{O_{2} }}^{in0} + I_{fc}^{0} K_{r} } \right)}}{{K_{{O_{2} }} T_{0} \tau_{{O_{2} }}^{0} }}} & { - \frac{{K_{r} T^{0} }}{{K_{{O_{2} }} T_{0} \tau_{{O_{2} }}^{0} }}} & 0 & {\frac{{T^{0} }}{{T_{0} K_{{O_{2} }} \tau_{{O_{2} }}^{0} }}} \\ 0 & 0 & {\frac{{ - T^{0} }}{{T_{0} \tau_{{h_{2} O}}^{0} }}} & { - \frac{{\left( {K_{{h_{2} O}} P_{{h_{2} O}}^{0} - 2I_{fc}^{0} K_{r} } \right)}}{{K_{{h_{2} O}} T_{0} \tau_{{h_{2} O}}^{0} }}} & {\frac{{2K_{r} T^{0} }}{{K_{{h_{2} O}} T_{0} \tau_{{h_{2} O}}^{0} }}} & 0 & 0 \\ {T_{ph} } & {T_{po} } & {T_{pw} } & {T_{pt} } & {T_{pi} } & {T_{pnh} } & {T_{pno} } \\ 0 & 0 & 0 & 0 & {\frac{{ - 1}}{{T_{el} }}} & 0 & 0 \\ {H_{y1} } & 0 & 0 & {H_{y2} } & {H_{y3} } & {H_{y4} } & 0 \\ 0 & {O_{x1} } & 0 & {O_{x2} } & {O_{x3} } & 0 & {O_{x4} } \\ \end{array} } \right] \\ B_{sys} & = \left[ {\begin{array}{*{20}c} 0 & 0 & 0 & 0 & {\frac{1}{{T_{el} }}} & {\frac{{2K_{r} K_{ihy} }}{{K_{{h_{2} }} }}} & {\frac{{K_{r} K_{iox} }}{{K_{{O_{2} }} }}} \\ \end{array} } \right]^{T} \\ \end{aligned}$$

As the system eigenvalues now become dependent on the PI controller gains, any change in the numerical values of these gains will modify the closed loop eigenvalues. So, there is every possibility that for a certain set of controller gains the closed loop eigenvalues as well as the system small signal dynamic response will give optimal response.

## Simulation results

To show the effectiveness of the proposed method of designing controllers for the SOFC system, the performance of the DE algorithm is compared with two other optimization techniques namely, PSO and IWO. Detail of the SOFC parameters used in the simulation is presented in the “Appendix”. Eigenvalue analysis and time domain based simulations along with two statistical tests are conducted to verify the effectiveness of DE. The details of the different cases considered for the time domain based simulation are listed in Table 2.

**Table 2:** Detail of different disturbances

Type of disturbance	Duration/Instants of applications	Magnitude of disturbance
Step	5.0 s onwards	30 A
Pulse	5–15 s	30 A
Staircase	5–15 s	30 A
	15–25 s	40 A
	25–35 s	50 A
	>35 s	0 A

### Controller tuning results

The result of the proposed tuning method is compared with those obtained by PSO and IWO. The above mentioned PI controller tuning procedure was carried out by a computer program coded in MATLAB. The programs were executed on a 2.50 GHz Intel Core i5 processor with 4 GB of random access memory (RAM). The parameters used for different algorithms are listed in Table 3. From practical point of view the controller gains should not be too high or too low and due to this fact the optimizer search space has been kept within some upper and lower limits which are presented in Table 4. This limited search space should certainly reduce the computational time taken by the optimizers.

**Table 3:** Parameters used for PSO, DE and IWO algorithm

Parameter	PSO	DE	IWO
Maximum population size	100	100	100
Maximum number of generations	2000	2000	2000
Stopping criteria (number of consecutive iterations with same value of objective function)	50	50	50
c1, cognitive acceleration coefficient	2	–	–
c2, social acceleration coefficient	0.01	–	–
MF, mutation factor	–	0.9	–
CR, crossover probability	–	0.2	–
Initial size of population	–	–	25
Maximum deviation, sdmax	–	–	5
Minimum deviation, sdmin	–	–	0
Nonlinear modulation index, n	–	–	2
Sigma_initial	–	–	1
Sigma_final	–	–	0.001

**Table 4:** Upper and lower bounds of the controller gains

Parameter	Lower bound	Upper bound
K_phy_	−7	−4
K_ihy_	−7	−4
K_pox_	−10	−7
K_iox_	−10	−7

### Eigenvalue analysis

For the statistical comparison, the developed code was run for 30 times for all the algorithms and the eigenvalues obtained for the best outcome are listed in Table 5. The eigenvalues related to the target variables are represented in italic format. Comparison of Table 5 with Table 1 reveals that under closed loop system two sets of complex eigenvalues are obtained and their real parts are more negative than the corresponding open loop eigenvalues which ensures improvement of system dynamic response. Specifically, the real parts of the open loop eigenvalues associated with hydrogen and oxygen partial pressures are −0.0343 and −0.3436; whereas the real part of corresponding closed loop eigenvalues with DE are −0.1782 and −0.8537, with PSO are −0.1782 and −0.8536, and with IWO are −0.1765 and −0.8501, respectively. However, as stated earlier, the other three eigenvalues associated with the temperature, water vapor partial pressure and SOFC current are unchanged even with the inclusion of the controllers. The optimized parameters for the best run of all the optimizers are listed in Table 6. Observation of Table 6 shows that both IWO and DE can obtain the optimized parameters within the search space but PSO reaches either the upper or the lower limit for all parameters.

**Table 5:** Eigenvalues obtained by PSO, DE and IWO (best from 30 runs)

Method	PSO	DE	IWO
Eigenvalues	−0.0171	−0.0171	−0.0171
	−*0.8537* + *0.4752i*	−*0.8536* + *0.4755i*	−*0.8501* + *0.5938i*
	−*0.8537* − *0.4752i*	−*0.8536* − *0.4755i*	−*0.8501* − *0.5938i*
	−*0.1782* + *0.3873i*	−*0.1782* + *0.3874i*	−*0.1765* + *0.4151i*
	−*0.1782* − *0.3873i*	−*0.1782* − *0.3874i*	−*0.1765* − *0.4151i*
	−0.0128	−0.0128	−0.0128
	−0.2000	−0.2000	−0.2000

**Table 6:** Optimized parameters by PSO, DE and IWO algorithms (best from 30 runs)

Parameter	PSO	DE	IWO
K_phy_	−7	−6.9994	−6.9247
K_ihy_	−4	−4.0002	−4.4760
K_pox_	−10	−9.9993	−9.9482
K_iox_	−7	−7.0012	−7.8852

### Time domain based simulation

**Fig. 7:**
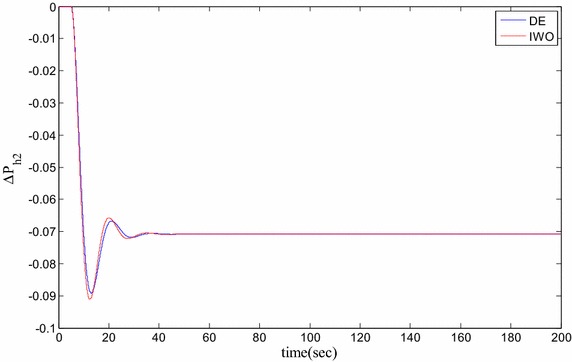
Response of hydrogen partial pressure for step load change

**Fig. 8:**
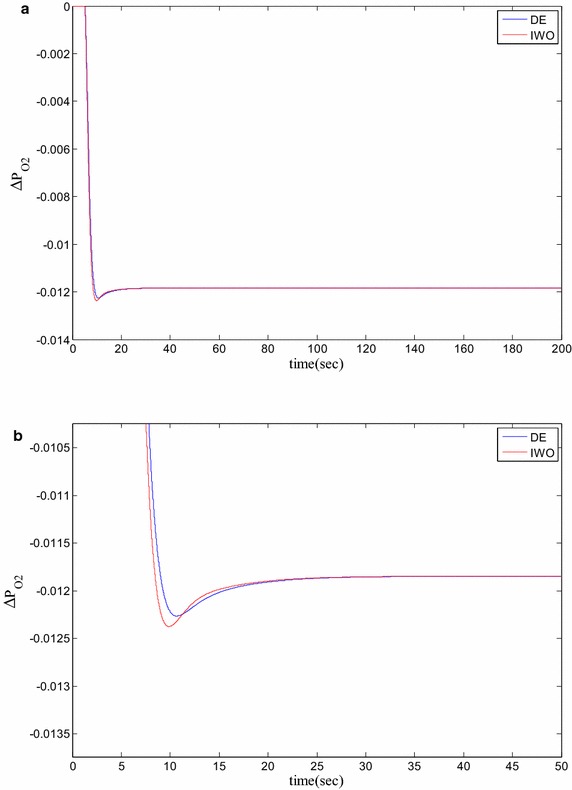
**a** Response of oxygen partial pressure for step load change, **b** zoomed view of the undershoot

**Fig. 9:**
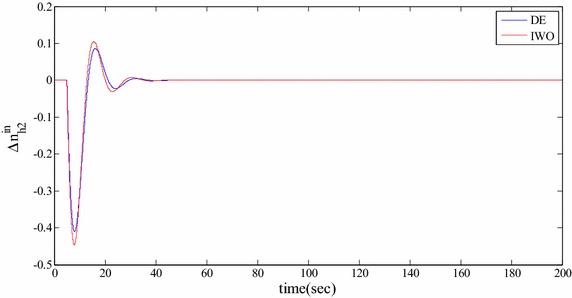
Response of hydrogen flow input for step load change

**Fig. 10:**
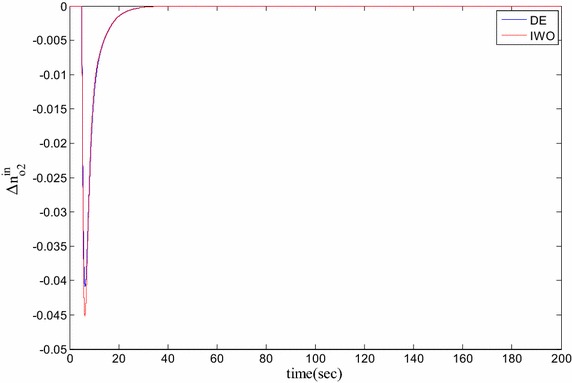
Response of oxygen flow input for step load change

**Fig. 11:**
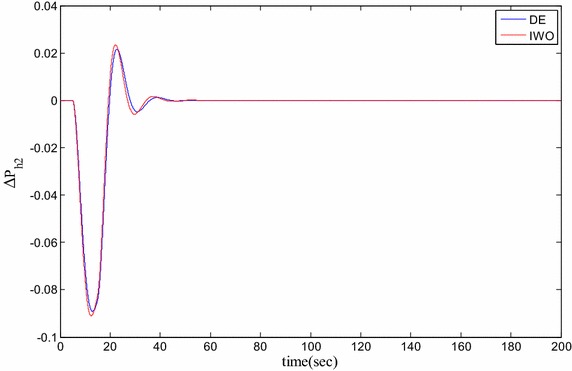
Response of hydrogen partial pressure for pulse disturbance

**Fig. 12:**
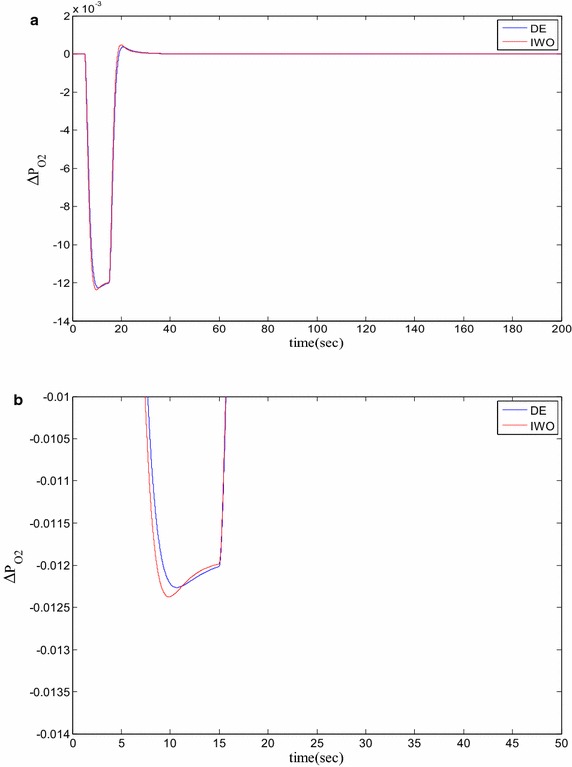
**a** Response of oxygen partial pressure for pulsed disturbance, **b** zoomed view of the undershoot of the response

**Fig. 13:**
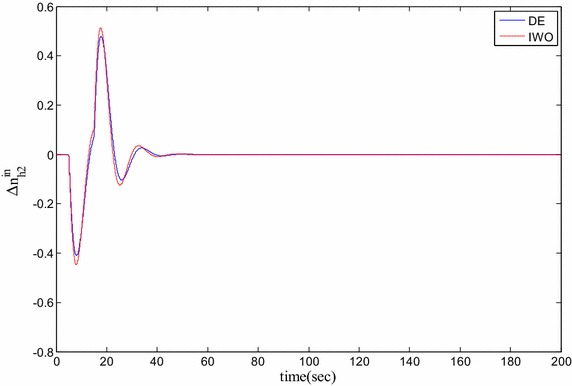
Response of hydrogen flow input for pulsed disturbance

**Fig. 14:**
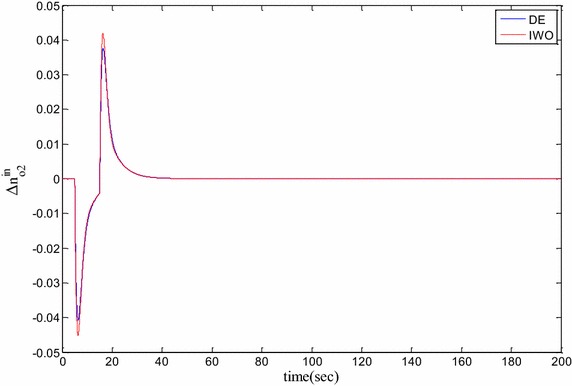
Response of oxygen flow input for pulsed disturbance

**Fig. 15:**
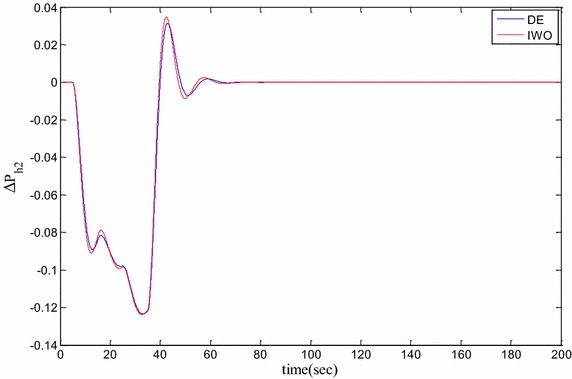
Response of hydrogen partial pressure for staircase type disturbance

**Fig. 16:**
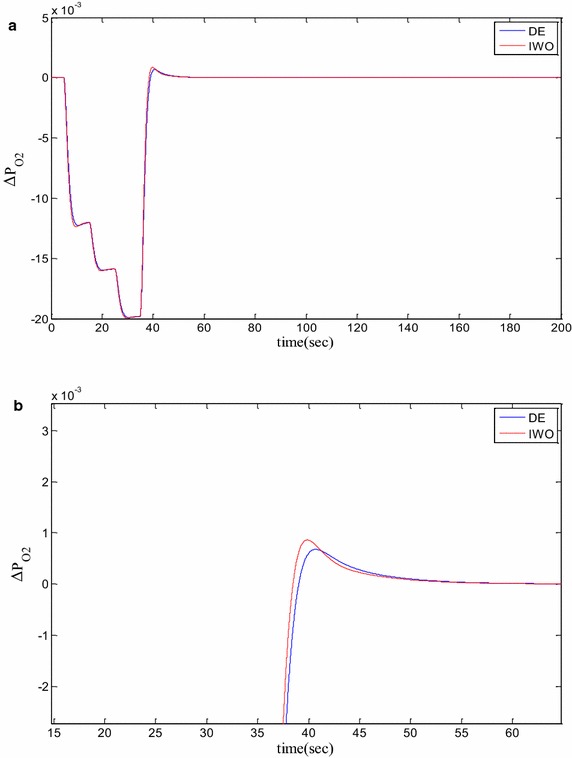
**a** Response of oxygen partial pressure for staircase type disturbance, **b** zoomed view of the overshoot of the response

**Fig. 17:**
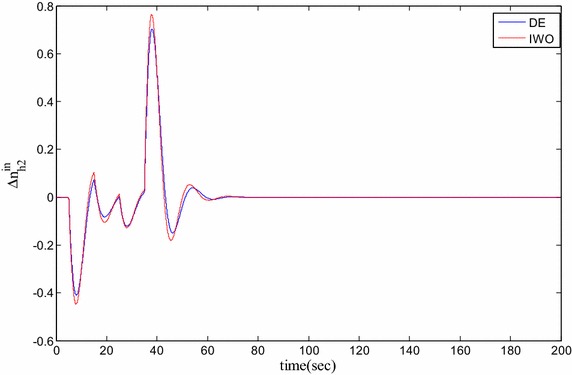
Response of hydrogen flow input for staircase type disturbance

**Fig. 18:**
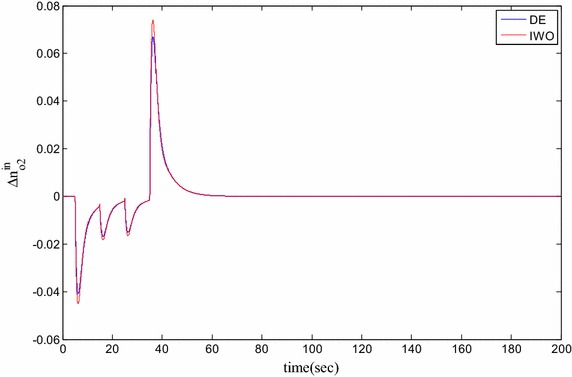
Response of oxygen flow input for staircase type disturbance

The performance of DE and IWO for different types of load variations are compared and the corresponding time domain simulations are presented in Figs. 7, 8, 9, 10, 11, 12, 13, 14, 15, 16, 17 and 18. As the optimized parameters obtained by PSO are almost identical to DE and the best fitness value reached by these two are same, the time domain responses are completely matched. So, the time domain responses of PSO are not included in this part. Figure 7 present the hydrogen partial pressure responses for step load change. The first undershoot and overshoot obtained by DE are found to be better than those obtained by IWO. Figure 8a presents the response of oxygen partial pressure for the step change in load. As the difference is not too vivid from this figure, a zoomed view of undershoot is given in Fig. 8b where the superiority of DE over IWO is clearly visible. The control inputs required for the step load change are presented in Figs. 9 and 10. The control efforts required by DE are found to have less overshoot/undershoot while the corresponding settling times are found almost identical.

The dynamic responses of the hydrogen and oxygen partial pressures for the pulse load variation are presented in Figs. 11 and 12 and the corresponding variations in the control inputs are shown in Figs. 13 and 14. The DE performance is found to be superior in this case, too. Finally, the responses of the hydrogen and oxygen partial pressures and the corresponding control inputs for staircase type load variations are presented in Figs. 15, 16, 17 and 18 which show the efficacy of DE over IWO once more.

#### Comparative study and non parametric statistical analysis

A comparative study of DE, PSO and IWO is presented in Table 7 in terms of elapsed time and best fitness value achieved in minimizing the eigenvalue based objective function. To make a fair comparison among the algorithms used, same number of generations (iterations) and same boundary limits are considered. Moreover, the stopping criterion for all algorithms is also set at a maximum number of generations. The optimizer will stop if it reaches the same fitness value for the maximum number of generation consecutively. Each algorithm is run for 30 times and the average running time is also reported. It is found from Table 7 that the best fitness value achieved by DE and PSO are exactly same (−2790.1) whereas the one obtained by IWO (−2787.7) is little worse. However, the average fitness value obtained by DE algorithm (−2790.0) is better than PSO (−2789.5) and IWO (−2781.1) for the 30 runs. With respect to the computational time, the average achieved by the DE algorithm is little inferior to IWO but far better than PSO. Hence, from overall observations of this Table it can be concluded that on an average the performance attained by DE is better than those obtained by PSO and IWO.

**Table 7:** Fitness value and time required by PSO, DE and IWO algorithms (30 runs)

Parameter	Best result			Average result			Worst result		
	PSO	DE	IWO	PSO	DE	IWO	PSO	DE	IWO
Elapsed time	2.0515	4.2550	5.8150	34.3702	8.1811	6.4268	79.3476	16.8045	7.3681
Best fitness value	−2790.1	−2790.1	−2787.7	−2789.5	−2790.0	−2781.1	−2788.4	−2789.8	−2771.3

To further investigate the conclusions obtained thus far, non-parametric statistical analysis of the data obtained from 30 independent test runs is performed using SPSS software. At first, the one sample Kolmogorov–Smirnov (KS) test is conducted where the null hypothesis, *H*_0_, assumes that the data sample fits normal distribution while the alternative hypothesis, *H*_1_, assumes that the data sample does not fit the normal distribution with a significance level of 0.05. From the analyses presented in Table 8 it is seen that none of the data sample is missed by the test as in all cases *N* = 30 samples are considered. The mean of the data sample shows that the DE algorithm outperforms the PSO and IWO in this regard. The value of standard deviation illustrates that the data samples for DE algorithm are much more adjacent to the best solution as compared to PSO and IWO which reflects higher stability. The *p* value (Asymp. Sig. 2-tailed) for DE algorithm is found as 0.015 which is lower than 0.05 and thus it can be said that the data sample for DE is significant enough to reject *H*_0_ and accept *H*_1_. On the other hand, the *p* values for PSO and IWO are greater than 0.05 which do not show enough significance to reject *H*_0_ and accept *H*_1_. Therefore, this test has 95 % confidence that the data set for DE is different than PSO and IWO.

**Table 8:** One sample Kolmogorov–Smirnov test results

	DE_fitness	PSO_fitness	IWO_fitness
N	30	30	30
Normal parameters			
Mean	−2790.02189	−2789.54599	−2781.05280
SD	.078069	.484365	3.460139
Most extreme differences			
Absolute	.285	.140	.094
Positive	.285	.140	.094
Negative	−.198	−.125	−.070
Kolmogorov–Smirnov Z	1.559	.769	.517
Asymp. Sig. (2-tailed)	.015	.595	.952

Next, the paired *t* test is run with the 30 samples to validate each algorithm distinctly and to discover further differences among them. The results for this test are shown in Table 9. The *H*_0_ in this case assumes that the means of the data sets are equal and *H*_1_ assumes the alternative statement that the means are unequal with a significance level of 0.05. As observed from Table 9, all the paired *p* values (Sig. 2-tailed) are less than 0.05 and thus show a significant level to reject *H*_0_. So, this study shows 95 % confidence level that the three pairs are significantly different from each other. Moreover, the negative correlation for each pair also suggests that the pairs are not related to one another. Thus, it can be concluded that the DE algorithm behaves differently in a statistical manner compared to other two and gives better performance.

**Table 9:** Paired sample *t* test results

Method	Paired differences				
	Mean	Correlation	T	df	Sig. (2-tailed)
DE-PSO	−.475895	−.196	−5.157	29	0.000
DE-IWO	−8.969086	−.047	−14.179	29	0.000
PSO-IWO	−8.493191	−.102	−13.133	29	0.000

## Conclusion

A simple PI controller based approach is proposed for improving the small signal dynamic response of a stand-alone SOFC plant where the controller parameters are tuned by the DE algorithm. Performance of DE is compared with those of PSO and IWO from different aspects. Eigenvalue based optimization is performed for the optimal tuning of the PI controller parameters. The superiority of eigenvalue based objective function over time domain based objective function lies in the fact that the computation time can be saved in the first instance. The results obtained by eigenvalue analysis show that the performance of DE and PSO are better than the IWO. This fact has been later justified by the minimum value of fitness function obtained by these algorithms. The lowest mean of the fitness function is obtained by the DE samples. Although the run time result shows that the IWO can reach to a minimum little faster than DE and much faster than PSO on an average, due to the inferiority of the minimum value of the fitness function obtained it fails to be regarded as the best optimizer. However, all the parameters obtained by PSO hit either the upper or the lower limit of the boundary. Three different types of load variations are considered for the time domain based results. It is found that the optimal parameters obtained by the proposed eigenvalue based objective function can successfully track all of these variations. As the minimum value of fitness function found by DE and PSO are exactly same, the time domain results for PSO are not reported. The non-parametric statistical test results show that the data sample obtained by these algorithms for 30 independent runs are not correlated and the smallest standard deviation is recorded for DE. In conclusion, considering all the aforementioned discussions, it can be said that the overall performance of DE is better than those of PSO and IWO for the study under consideration.

## Appendix

### SOFC data

**Table:**

*E*_0_ = 1.28 V	*R* = 8.3146 J/mol-°C	*F* = 96,487 C/mol	*N*_0_ = 384
*T*_0_ = 923 °C	*T*_*ref*_ = 25 °C	*T*_2_ = 25 °C	$\Delta \hat{H}_{r}^{0}$ = −2.4183 kJ/mol
$K_{{h_{2} }}$ = 8.43e−4 kmol/(atm-s)	$K_{{O_{2} }}$ = 2.52e−3 kmol/(atm-s)	$K_{{h_{2} O}}$ = 2.81e−4 kmol/(atm-s)	$\tau_{{h_{2} }}^{0}$ = 26.1 s
$\tau_{{O_{2} }}^{0}$ = 2.91 s	$\tau_{{h_{2} O}}^{0}$ = 78.3 s	*m*_*e*_ = 1.1 kg	$\bar{C}_{p}$ = 1e4
*r*_0_ = 0.126 ohms	*α* = −2870	*T*_*in*_ = 900 °C	

### Linearizing constants

$$\begin{aligned} H_{1} & = dH_{2} \frac{{T_{2}^{4} }}{4} + cH_{2} \frac{{T_{2}^{3} }}{3} + bH_{2} \frac{{T_{2}^{2} }}{2} + aH_{2} T_{2} - \left( {dH_{2} \frac{{T^{4} }}{4} + cH_{2} \frac{{T^{3} }}{3} + bH_{2} \frac{{T^{2} }}{2} + aH_{2} T} \right) \\ O_{1} & = dO_{2} \frac{{T_{2}^{4} }}{4} + cO_{2} \frac{{T_{2}^{3} }}{3} + bO_{2} \frac{{T_{2}^{2} }}{2} + aO_{2} T_{2} - \left( {dO_{2} \frac{{T^{4} }}{4} + cO_{2} \frac{{T^{3} }}{3} + bO_{2} \frac{{T^{2} }}{2} + aO_{2} T} \right) \\ W_{1} & = dH_{2O} \frac{{T_{2}^{4} }}{4} + cH_{2O} \frac{{T_{2}^{3} }}{3} + bH_{2O} \frac{{T_{2}^{2} }}{2} + aH_{2O} T_{2} - \left( {dH_{2O} \frac{{T^{4} }}{4} + cH_{2O} \frac{{T^{3} }}{3} + bH_{2O} \frac{{T^{2} }}{2} + aH_{2O} T} \right) \\ N_{1} & = dN_{2} \frac{{T_{2}^{4} }}{4} + cN_{2} \frac{{T_{2}^{3} }}{3} + bN_{2} \frac{{T_{2}^{2} }}{2} + aN_{2} T_{2} - \left( {dN_{2} \frac{{T^{4} }}{4} + cN_{2} \frac{{T^{3} }}{3} + bN_{2} \frac{{T^{2} }}{2} + aN_{2} T} \right) \\ \end{aligned}$$

$$\begin{aligned} N_{12} & = dN_{2} \frac{{T^{4} }}{4} + cN_{2} \frac{{T^{3} }}{3} + bN_{2} \frac{{T^{2} }}{2} + aN_{2} T \\ H_{12} & = dH_{2} \frac{{T^{4} }}{4} + cH_{2} \frac{{T^{3} }}{3} + bH_{2} \frac{{T^{2} }}{2} + aH_{2} T \\ O_{12} & = dO_{2} \frac{{T^{4} }}{4} + cO_{2} \frac{{T^{3} }}{3} + bO_{2} \frac{{T^{2} }}{2} + aO_{2} T \\ W_{12} & = dH_{2O} \frac{{T^{4} }}{4} + cH_{2O} \frac{{T^{3} }}{3} + bH_{2O} \frac{{T^{2} }}{2} + aH_{2O} T \\ \end{aligned}$$

$$\begin{aligned} G_{1h} & = \frac{{I_{fc}^{0} N_{0} RT^{0} }}{{2000FP_{{h_{2} }}^{0} }}\quad G_{2O} = \frac{{I_{fc}^{0} N_{0} RT^{0} }}{{4000FP_{{O_{2} }}^{0} }}\quad G_{3w} = \frac{{I_{fc}^{0} N_{0} RT^{0} }}{{2000FP_{{h_{2} O}}^{0} }} \\ Z_{1} & = \frac{1}{{\bar{C}_{p} m_{e} }}\quad M_{1} = \left( {T_{2} - T^{0} } \right)\quad T_{ph} = - M_{1} K_{{h_{2} }} \left( {H_{1} + G_{1h} } \right)Z_{1} \\ T_{po} & = - M_{1} K_{{O_{2} }} \left( {O_{1} + G_{2O} } \right)Z_{1} \quad T_{pw} = - M_{1} K_{{h_{2} O}} \left( {W_{1} - G_{3w} } \right)Z_{1} \\ \end{aligned}$$

$$\begin{aligned} T_{pr} & = \frac{{I_{fc}^{0} \left( {N_{0} \left( {\frac{R}{2F}\ln \frac{{P_{{h_{2} }}^{0} P_{{O_{2} }}^{{0^{0.5} }} }}{{P_{{h_{2} O}}^{0} }} - 2.52e^{ - 4} } \right) - \frac{{I_{fc}^{0} \alpha r^{0} e^{{\alpha \left( {\frac{1}{{T_{0} }} - \frac{1}{{T^{{_{0} }} }}} \right)}} }}{{T^{{0^{2} }} }}} \right)}}{1000} \\ T_{{pt}} & = \dot{n}_{{n_{2} }}^{{out}} \left( {N_{1} + M_{1} \left( {\dot{n}_{{n_{2} }}^{{out}} N_{{12}} + \dot{n}_{{h_{2} }}^{{out}} H_{{12}} + \dot{n}_{{h_{2} O}}^{{out}} W_{{12}} + \dot{n}_{{O_{2} }}^{{out}} O_{{12}} } \right)} \right. \\ & \quad \left. { - Tpr + \dot{n}_{{h_{2} }}^{{out}} H_{1} + \dot{n}_{{h_{2} O}}^{{out}} W_{1} + \dot{n}_{{O_{2} }}^{{out}} O_{1} } \right)Z_{1} \\ T_{pi} & = - \left( {\frac{{N_{0} \left( {\frac{{RT^{0} }}{2F}\ln \frac{{P_{{h_{2} }}^{0} P_{{O_{2} }}^{{0^{0.5} }} }}{{P_{{h_{2} O}}^{0} }} - 2.52e^{ - 4} T^{0} + 1.2586} \right)}}{1000} + \frac{{K_{r} \Delta \hat{H}_{r}^{0} }}{500} - \frac{{I_{fc}^{0} r^{0} e^{{\alpha \left( {\frac{1}{{T_{0} }} - \frac{1}{{T^{{_{0} }} }}} \right)}} }}{500}} \right)Z_{1} \\ \end{aligned}$$

$$\begin{aligned} H_{t2} & = dH_{2} \frac{{T_{2}^{4} }}{4} + cH_{2} \frac{{T_{2}^{3} }}{3} + bH_{2} \frac{{T_{2}^{2} }}{2} + aH_{2} T_{2} - \left( {dH_{2} \frac{{T_{in}^{4} }}{4} + cH_{2} \frac{{T_{in}^{3} }}{3} + bH_{2} \frac{{T_{in}^{2} }}{2} + aH_{2} T_{in} } \right) \\ O_{t2} & = dO_{2} \frac{{T_{2}^{4} }}{4} + cO_{2} \frac{{T_{2}^{3} }}{3} + bO_{2} \frac{{T_{2}^{2} }}{2} + aO_{2} T_{2} - \left( {dO_{2} \frac{{T_{in}^{4} }}{4} + cO_{2} \frac{{T_{in}^{3} }}{3} + bO_{2} \frac{{T_{in}^{2} }}{2} + aO_{2} T_{in} } \right) \\ T_{pnh} & = - (T_{in} - T_{ref} )H_{t2} Z_{1} \quad T_{pno} = - (T_{in} - T_{ref} )O_{t2} Z_{1} \\ \end{aligned}$$

$$\begin{aligned} H_{y1} & = K_{ihy} - \frac{{K_{phy} T^{0} }}{{T_{0} \tau_{{H_{2} }}^{0} }}\quad H_{y2} = \frac{{ - \left( {K_{phy} \left( {K_{{h_{2} }} P_{{h_{2} }}^{0} - n_{{h_{2} }}^{in0} + 2K_{r} I_{fc}^{0} } \right)} \right)}}{{K_{{h_{2} }} T_{0} \tau_{{H_{2} }}^{0} }}\quad H_{y3} = \frac{{ - \left( {2K_{phy} K_{r} T^{0} } \right)}}{{K_{{h_{2} }} T_{0} \tau_{{H_{2} }}^{0} }} \\ H_{y4} & = \frac{{K_{phy} T^{0} }}{{K_{{h_{2} }} T_{0} \tau_{{H_{2} }}^{0} }}\quad O_{x1} = K_{iox} - \frac{{K_{pox} T^{0} }}{{T_{0} \tau_{{O_{2} }}^{0} }}\quad O_{x2} = \frac{{ - \left( {K_{pox} \left( {K_{{O_{2} }} P_{{O_{2} }}^{0} - n_{{O_{2} }}^{in0} + K_{r} I_{fc}^{0} } \right)} \right)}}{{K_{{O_{2} }} T_{0} \tau_{{O_{2} }}^{0} }} \\ O_{x3} & = \frac{{ - \left( {K_{pox} K_{r} T^{0} } \right)}}{{K_{{O_{2} }} T_{0} \tau_{{O_{2} }}^{0} }}\quad O_{x4} = \frac{{K_{pox} T^{0} }}{{K_{{O_{2} }} T_{0} \tau_{{O_{2} }}^{0} }} \\ \end{aligned}$$

## References

[CR1] Abido M (2002). Optimal design of power-system stabilizers using particle swarm optimization. IEEE Trans Energy Convers.

[CR2] Abou El Ela AA, Abido MA, Spea SR (2009). Optimal power flow using differential evolution algorithm. Electr Eng.

[CR4] Arriagada J (2002). Artificial neural network simulator for SOFC performance prediction. J Power Sources.

[CR5] Bhuyan KC, Sao SK, Mahapatra K (2013). An FPGA based controller for a SOFC DC-DC power system. Adv Power Electron.

[CR6] Bove R, Lunghi P, Msammes N (2005). SOFC mathematic model for systems simulations, Part 2: definition of an analytical model. Int J Hydrogen Energy.

[CR7] Campanari S, Iora P (2004). Definition and sensitivity analysis of a finite volume SOFC model for a tubular cell geometry. J Power Sources.

[CR8] Choudhury A, Chandra H, Arora A (2013). Application of solid oxide fuel cell technology for power generation—a review. Renew Sustain Energy Rev.

[CR9] Chowdhury A (2011). Automatic clustering based on invasive weed optimization algorithm. Swarm, evolutionary, and memetic computing.

[CR10] Coker AK (1995) Physical property of liquids and gases. Fortran programs for chemical process design, analysis, and simulation, pp. 103–149. doi:10.1016/b978-088415280-4/50003-0

[CR11] Das T, Slippey A (2010) Observer based transient fuel utilization control for solid oxide fuel cells. In: ASME 2010 dynamic systems and control conference, volume 1. doi:10.1115/dscc2010-4182

[CR12] Du W (2012). Effect of grid-connected solid oxide fuel cell power generation on power systems small-signal stability. IET Renew Power Gener.

[CR13] Ghilani CD (2010). Adjustment computations: spatial data analysis.

[CR14] Jacobsen LT, Spivey BJ, Hedengren JD (2013) Model predictive control with a rigorous model of a solid oxide fuel cell. In: 2013 American control conference. doi:10.1109/acc.2013.6580409

[CR15] Kamel RM, Chaouachi A, Nagasaka K (2013). Three control strategies to improve the microgrid transient dynamic response during isolated mode: a comparative study. IEEE Trans Ind Electron.

[CR16] Li YH, Choi SS, Rajakaruna S (2005). An analysis of the control and operation of a solid oxide fuel-cell power plant in an isolated system. IEEE Trans Energy Convers.

[CR17] Li Y (2012). Data-driven nonlinear control of a solid oxide fuel cell system. J Cent South Univ.

[CR18] Nehter P (2006). Two-dimensional transient model of a cascaded micro-tubular solid oxide fuel cell fed with methane. J Power Sources.

[CR19] Padullés J, Ault G, McDonald J (2000). An integrated SOFC plant dynamic model for power systems simulation. J Power Sources.

[CR20] Pepermans G (2005). Distributed generation: definition, benefits and issues. Energy Policy.

[CR21] Qin AK, Huang VL, Suganthan PN (2009). Differential evolution algorithm with strategy adaptation for global numerical optimization. IEEE Trans Evol Comput.

[CR22] Recknagle K (2003). Three-dimensional thermo-fluid electrochemical modeling of planar SOFC stacks. J Power Sources.

[CR3] Sanchez-Gasca J et al (2007) Small signal stability and power system oscillations. Electrical engineering handbook. doi:10.1201/9781420009248.ch

[CR23] Sedghisigarchi K, Feliachi A (2004). Dynamic and transient analysis of power distribution systems with fuel cells—Part I: fuel-cell dynamic model. IEEE Trans Energy Convers.

[CR24] Vesterstrom J, Thomsen R (2004) A comparative study of differential evolution, particle swarm optimization, and evolutionary algorithms on numerical benchmark problems. In: Proceedings of the 2004 congress on evolutionary computation (IEEE Cat. No. 04TH8753). doi:10.1109/cec.2004.1331139

[CR25] Wang S-K, Chiou J-P, Liu C-W (2009). Parameters tuning of power system stabilizers using improved ant direction hybrid differential evolution. Int J Electr Power Energy Syst.

[CR26] Wu X-J (2008). Nonlinear modeling of a SOFC stack based on ANFIS identification. Simul Model Pract Theory.

[CR27] Wu X (2008). Dynamic modeling of SOFC based on a T–S fuzzy model. Simul Model Pract Theory.

[CR28] Wu X-J (2009). A hybrid experimental model of a solid oxide fuel cell stack. J Fuel Cell Sci Technol.

